# Factors associated with delirium in a real-world acute-care setting: analysis considering the interdependence of clinical variables with the frailty syndrome

**DOI:** 10.1007/s41999-024-00934-x

**Published:** 2024-02-08

**Authors:** Andrea Ticinesi, Alberto Parise, Davide Delmonte, Chiara Coppi, Beatrice Prati, Nicoletta Cerundolo, Angela Guerra, Antonio Nouvenne, Tiziana Meschi

**Affiliations:** 1 Department of Medicine and Surgery, University of Parma, Via Antonio Gramsci 14, 43126 Parma, Italy; 2 Geriatric-Rehabilitation Department, Azienda Ospedaliero-Universitaria di Parma, Via Antonio Gramsci 14, 43126 Parma, Italy; 3 Institute of Materials for Electronics and Magnetism, National Research Council (CNR), Parco Area delle Scienze 7/A, 43124 Parma, Italy; 4 Doctoral School in Material Science, Department of Chemistry, Life Science and Environmental Sustainability, University of Parma, Parco Area delle Scienze 7/A, 43124 Parma, Italy

**Keywords:** Acute cerebral dysfunction, Cognitive impairment, Frailty, Geriatric emergency medicine, Delirium

## Abstract

**Purpose:**

Delirium risk assessment in the acute-care setting generally does not account for frailty. The objective of this retrospective study was to identify factors associated with delirium, considering the interdependency of clinical variables with frailty syndrome in complex older patients.

**Methods:**

The clinical records of 587 participants (248 M, median age 84) were reviewed, collecting clinical, anamnestic and pharmacological data. Frailty syndrome was assessed with the Clinical Frailty Scale (CFS). Delirium was the main study endpoint. The correlations of the considered anamnestic and clinical variables with delirium and its subtypes were investigated selecting only those variables not showing a high overlap with frailty. Correlations associated with a 25% excess of frequency of delirium in comparison with the average of the population were considered as statistically significant.

**Results:**

Delirium was detected in 117 (20%) participants. The presence of one among age > 85 years old, CFS > 4 and invasive devices explained 95% of delirium cases. The main factors maximizing delirium incidence at the individual level were dementia, other psychiatric illness, chronic antipsychotic treatment, and invasive devices. The coexistence of three of these parameters was associated with a peak frequency of delirium, ranging from 57 to 61%, mostly hypoactive forms.

**Conclusions:**

In acute-care wards, frailty exhibited a strong association with delirium during hospitalization, while at the individual level, dementia and the use of antipsychotics remained important risk factors. Modern clinical prediction tools for delirium should account for frailty syndrome.

**Supplementary Information:**

The online version contains supplementary material available at 10.1007/s41999-024-00934-x.

## Key summary points

**Aim:**

This study investigated factors associated with delirium in an acute-care setting, considering the interdependency of several clinical parameters with the frailty syndrome.

**Findings:**

In acute care, delirium was mainly present in participants with at least one among age > 85 years old, Clinical Frailty Scale > 4, and use of invasive devices. At the individual level, dementia, other psychiatric illnesses and use of antipsychotics were the greatest risk factors for delirium.

**Message:**

The evaluation of a limited number of clinical parameters, including frailty, can help clinicians identify older patients at high risk of delirium in acute-care wards with a high level of accuracy.

**Supplementary Information:**

The online version contains supplementary material available at 10.1007/s41999-024-00934-x.

## Introduction

Delirium, an acute disorder of attention and cognition with fluctuating course, is one of the commonest complications occurring in older patients urgently admitted to hospital [1]. Delirium is particularly frequent in internal medicine and geriatric acute-care wards, and implies a significant burden of illness, mortality and costs [2].

Several factors predisposing to delirium have been identified in previous studies, including dementia, functional impairment, visual and hearing problems, depression and age ≥ 75 years old [1]. In patients with one or more of these conditions, delirium can be also triggered by precipitating factors associated with acute illness and hospital care, including renal failure, hyperglycemia, hypoalbuminemia, metabolic acidosis, hypoxemia, infection, use of drugs with anticholinergic action, insertion of bladder catheters and surgery with substantial blood loss [1]. Acute illness severity and prolongation of hospital stay are also well-established factors associated with delirium [3].

Some researchers have proposed multiparametric tools for predicting delirium onset in hospital patients [4, 5]. Despite their good performance, these tools require the collection of several anamnestic, physiologic and laboratory parameters, and are not always centered on the characteristics of geriatric patients.

Recently, pre-clinical and clinical evidences have highlighted that delirium shares important pathophysiological mechanisms with the frailty syndrome [6]. According to the current international consensus, frailty is defined as a state of increased vulnerability to stressors typical of ageing, with impaired homeostasis and ability to return to the status quo ante, increasing the odds of adverse events and loss of functional independence [7] Frailty diagnosis is generally based on the assessment of its typical phenotype, formalized in the Fried criteria (presence of at least three among unintentional weight loss, muscle weakness, exhaustion, slowness and low activity) [8]. Other tools based on the deficit accumulation model of frailty, such as the Frailty Index (FI) or its quicker version called Clinical Frailty Scale (CFS), are also very popular in clinical practice, especially in the acute care setting. In fact, they can be easily calculated from common clinical data collected upon admission [8]. These tools measure frailty and its severity by considering the ratio between the number of deficits or health alterations present in each patient and the number of conditions assessed during routine clinical examination and geriatric assessment [9], and show a significant correlation with Fried criteria [10, 11]. Despite the variability of tools used to assess both delirium and frailty in clinical studies, two independent meta-analyses have recently recognized frailty as an independent risk factor for delirium in older individuals [12, 13], especially in the acute-care setting [14].

Interestingly, several of the conditions previously identified as independent risk factors for delirium are assessed in tools used for frailty evaluation, such as FI or CFS [15]. Both FI and CFS can predict delirium in acute older patients and are increasingly used also outside the geriatric setting [13, 14, 16, 17]. Thus, the stratification of delirium risk may be facilitated by routine assessment of frailty, because several clinical conditions traditionally considered as risk factors for delirium may be interdependent with frailty.

The aim of this retrospective study was to identify factors independently associated with delirium in a group of older patients urgently admitted to an acute-care internal medicine ward, considering the interdependency between several anamnestic conditions and frailty.

## Methods

### Study setting and population

The study was conducted in an acute-care high-turnover internal medicine unit of the Geriatric-Rehabilitation Department of a 1200-bed teaching hospital in Northern Italy (Parma University-Hospital) [18], before the emergence of the coronavirus disease-19 (COVID-19) pandemic.

The clinical records of all patients admitted between February and April 2019 were checked for study inclusion, and only patients urgently admitted after an Emergency Department (ED) visit were selected. Excluded from the study were all those patients with addiction, psychiatric comorbidities other than dementia, or lacking data on delirium assessment on their clinical records.

### Data collection

We retrospectively collected data on age, sex, daytime and weekday of ward admission, ED boarding time, acute illness that caused hospitalization, chronic comorbidities, frailty, number and pharmacologic class of each medication taken chronically before admission, baseline serum lab tests, use of drugs with anticholinergic activity, use of invasive devices (urinary catheters, nasogastric tubes, central venous lines) during hospital stay. All these parameters were considered as exposure variables.

The presence and severity of multimorbidity was assessed through the count of chronic illnesses and calculation of the Cumulative Illness Rating Scale (CIRS) Comorbidity Score (CIRS-CS), defined as the sum of the scores, ranking from 0 to 4, assigned to each of fourteen items corresponding to the main body organs and systems in accordance with the presence of chronic illnesses and their severity, and the CIRS Severity Index (CIRS-SI), corresponding to the number of items ranking 3 or 4 [19].

Frailty was assessed and measured with the CFS, a scale ranging from 1 (very fit) to 9 (very poor health status with terminal illness) [20], which has proven very accurate for predicting functional decline and mortality in several settings of geriatric care [21].

The diagnosis of delirium was made on a clinical basis, in compliance with the DSM-5 (Diagnostic and Statistical Manual of Mental Disorders) criteria. In those participants with a strong clinical suspicion of delirium, the Confusion Assessment Method (CAM) scale was administered during hospital stay [22], and was used to corroborate the diagnosis of delirium for the purposes of the present study. In all other participants, DSM-5 criteria for delirium were checked retrospectively from case notes on medical records, in compliance with the methodology also used in other studies [23–26]. If a patient fulfilled DSM-5 criteria onward admission, (s)he was classified as having prevalent delirium. Conversely, incident delirium was defined as delirium of novel onset during the hospital stay. Delirium subtypes (i.e., hyperactive or hypoactive) were also identified from daily notes on clinical records, similar to the methodology used in COVID-19 studies [27, 28]. Participants with restlessness, agitation, wandering, increased alertness and hallucinations were classified as having hyperactive delirium, while participants with reduced motor activity, lethargy, withdrawal and inappropriate drowsiness as having hypoactive delirium.

Finally, the outcome of hospitalization (death, discharge to nursing home or patient’s own home) was considered, in order to verify the association between delirium and mortality.

### Statistical analyses

For descriptive purposes, the clinical parameters and outcomes available in the dataset were presented as median and interquartile range (IQR) or as percentages, according to the type of variable.

Statistical distribution of different discrete parameters of the patient (including age, CFS, number of chronic illnesses, number of drugs taken before admission) was then studied via binomial-Poisson statistics and correlated with delirium (primary endpoint variable) with linear regression through least squares method, estimating the *R*^2^ as standard marker of the fitting quality (*R*^2^* → *0 bad interpolation, *R*^2^* → *1 good interpolation). Delirium was investigated as a function of the clinical characteristics of the patient, particularly by discriminating three categories of correlations:

1. intrinsic conditions, including age, gender, CFS, number and type of chronic illnesses, CIRS-CS, CIRS-SI, number and type of drugs, reasons for hospitalization;
2. incident conditions, focusing on the use of devices during hospital stay;
3. hospitalization outcome, including death, transfer to the nursing home, or domicile.

For the purposes of the present analysis, non-binary discrete variables were reduced to binary logics creating a proper threshold for each of them: in this approach the value 1 was assigned when the parameter is greater than a clinically significant threshold, and the value 0 if lower. To identify which parameters impacted the most on delirium, each exposure variable was individually cross-correlated with delirium. The whole dataset was then filtered by selecting those variables associated with a frequency of delirium (considering incident and prevalent forms altogether) ≥ 25%, corresponding to an excess of at least 25% with respect to the mean value detected in the entire population. This allowed us to discriminate those correlations showing an excess of cases well above the error of statistical fluctuations around the mean value and to obtain a limited number of exposure variables showing a strong correlation with delirium (category 1).

Consequently, interdependence between such selected parameters was comprehensively analyzed, to evaluate the degree of independence of each possible couple of variables by means of standard set theory. In this approach, an empiric measurement of interdependence is given by the value assumed by the fraction of intersection between the subset of patients with one parameter and the subset of patients with another parameter vs. the small subset between the two:

1 $$C_{{{\text{ID}}}} = \, S_{{{\text{PAR}}1}} \cap \, S_{{{\text{PAR}}2}} /{\text{min }}(S_{{{\text{PAR}}1}} ,S_{{{\text{PAR}}2}} )$$

where *C*_*ID*_ is the interdependence coefficient empiric value, S_PAR1_ is the subset of patients displaying a generic parameter #1 (PAR1) and *S*_PAR2_ is the subset of patients displaying the presence of a generic parameter #2 (PAR2).

These results, gathered in the matrix shown in Supplementary Table 1, allowed us to identify some variables, among those selected with the previous protocol, with strong correlation among them. One notable example was the interdependence coefficient calculated for *PAR1* = *Dementia* and *PAR2* = *frailty*, identified by CFS > 4: almost all the patients affected by dementia belonged to the class of frailty. Therefore, these two variables were not considered simultaneously, but rather independently of one another.

Standing this assumption, we proceeded by selecting parameters of category (1) displaying the largest correlation with delirium, and adding one by one the parameters of categories (2) and (3) in a multi-correlated approach looking at those correlations maximizing the incidence of delirium in the subpopulation of participants showing the characteristics defined by selected parameters and the coverage of the total number of patients with delirium episodes.

Analogous statistical approach was used to evaluate the effects of intrinsic conditions, incident conditions and hospitalization outcomes as a function of the different types of delirium (incident or prevalent, hyperactive or hypoactive). In these cases, the analyses and outputs obtained for delirium of all types were used as the benchmark to estimate further polarizations and/or enhancements of the correlations emerged in the general case. A similar approach was also adopted for the identification of factors associated with delirium subtypes.

The statistical analyses were performed via specific user-made codes developed by means of the Matlab Mathworks platform [29].

### Ethical statement

The study protocol was approved by the competent Ethics Committee (Comitato Etico dell’Area Vasta Emilia Nord, Regione Emilia-Romagna) under the ID 84/2019/OSS/AOUPR. Due to the retrospective design of the study, informed consent was collected in written form only whenever possible. In other cases, informed consent collection was waived. The study was conducted in compliance with the Declaration of Helsinki and its later amendments.

## Results

The studied population included 587 patients (248 males and 339 females), with a median age of 84 years old (IQR 79–91). Among them, 117 (20%) presented delirium during hospital stay. A comparison of the reasons for admission and clinical features between patients with and without delirium is presented in Table 1 and Supplementary Table 2. Namely, patients with delirium were older (median age 87, IQR 82–92, vs 83, IQR 78–88 years old), with a similar number of comorbidities but more severe frailty (median CFS 6, IQR 5–7, vs 5, IQR 4–6). Pharmacologic treatment with atypical antipsychotics (25% vs 9%) and anti-epileptic drugs (14% vs 7%) on admission was more common in patients with delirium.

**Table 1:** Comparison of the main clinical characteristics on admission of patients who presented features of delirium and those who did not

Parameter	Patients with delirium (n = 117)	Patients without delirium (n = 470)
**General characteristics**		
Age, years	87 (82–92)	83 (78–88)
Females, %	65	56
Emergency Department boarding time, hours	26 (20–37)	26 (20–34)
Weight, kg	58 (51–70)	66 (56–78)
BMI, kg/m^2^	24 (22–26)	26 (22–28)
**Personal history**		
Chronic illnesses, number	5 (3–7)	5 (3–7)
Hypertension, %	67	70
Cardiomyopathy, %	36	50
Arrythmia, %	38	36
Anemia, %	14	28
COPD, %	24	31
Diabetes, %	22	29
Obesity, %	4	9
Dyslipidemia, %	13	21
Parkinsonism, %	13	7
Previous stroke, %	17	13
CKD, %	13	16
Cancer, %	15	29
Dementia, %	55	23
Psychiatric diseases other than dementia, %	28	15
Peripheral artery disease, %	31	29
Liver disease, %	12	13
Gastrointestinal disease, %	19	26
CIRS Comorbidity Score	12 (9–15)	13 (9–6)
CIRS Severity Index	2 (0–3)	2 (1–3)
Clinical Frailty Scale	6 (5–7)	5 (4–6)
Chronic drug treatments, number	6 (4–8)	7 (4–9)
**Laboratory parameters**		
Hemoglobin, g/dl	11.9 (10.5–13.4)	11.7 (10.1–13.2)
CRP, mg/L	64 (26–135)	54 (16–119)
**Main reasons for admission**		
Pneumonia, %	24	10
Heart failure, %	14	14
Stroke, %	5	1
Falls excluding syncope, %	8	3

Data are presented as median and interquartile range or percentage

*BMI* body mass index, *COPD* chronic obstructive pulmonary disease, *CKD* chronic kidney disease, *CIRS* cumulative illness rating scale, *CRP* C-reactive protein

**Fig. 1:**
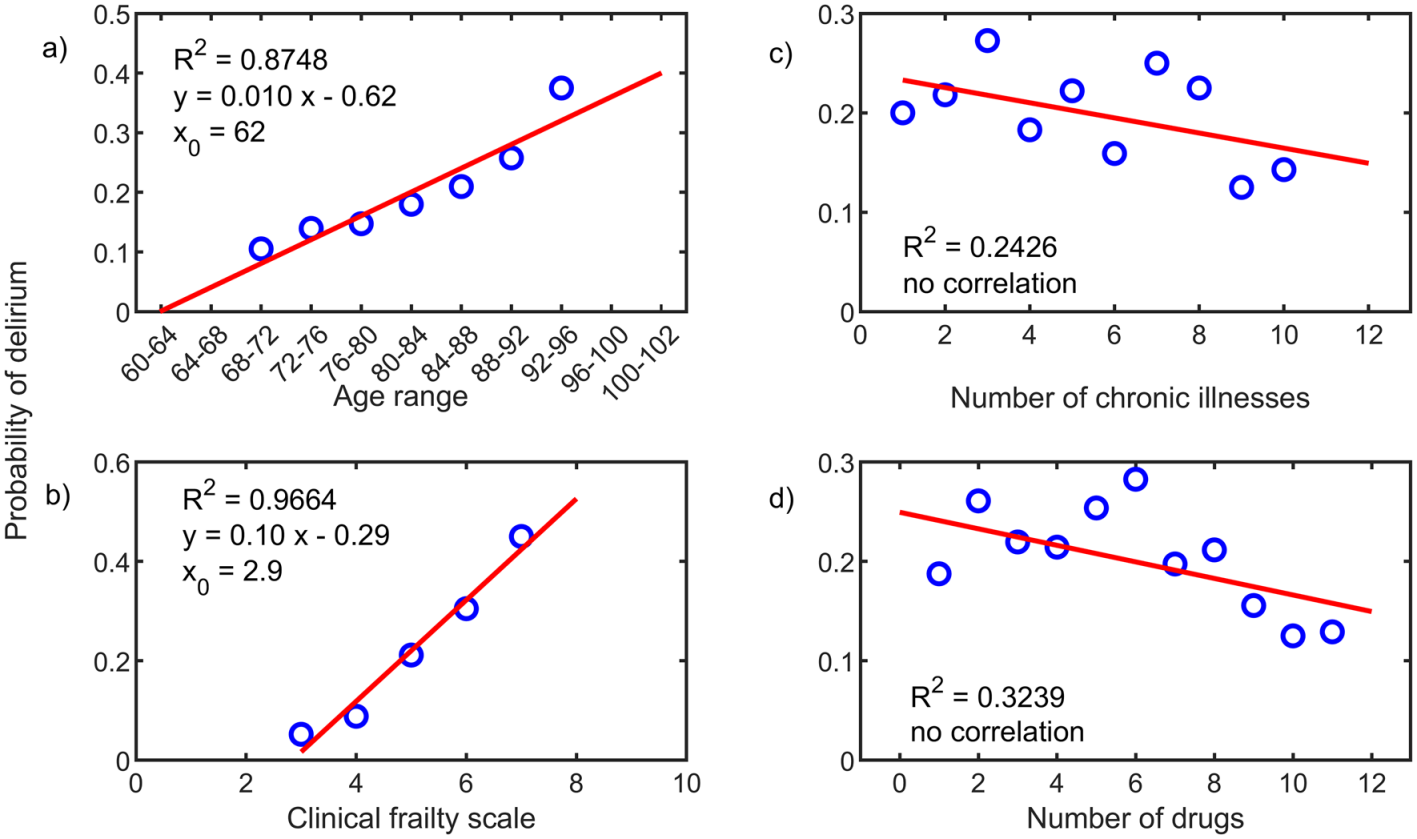
Correlation analysis between the presence of delirium during hospitalization and the main exposure variables considered in the study: **a** age; **b** clinical frailty scale (CFS); **c** number of chronic illnesses; **d** number of drugs. In all the subplots the R^2^ value is reported. Where the correlation is observed (**a**) and (**b**), the linear fit equation is shown, highlighting the x_0_ value in which the onset of delirium arises

Age (R^2^ = 0.874, β = 0.010 ± 0.002), with the onset of correlation at 62 years old, and CFS (R^2^ = 0.966, β = 0.10 ± 0.03), with the onset of correlation for scores equal to 3 or higher, were linearly correlated with the presence of delirium during hospital stay (Fig. 1). Patients with CFS > 4, representing 57% of the population, determined 85% of the total cases of delirium. Consequently, the delirium incidence was 34% for participants with CFS > 5 and 38% for those with CFS > 6, with a remarkable excess of frequency in comparison with the mean value of the entire population (70% and 90% vs. 20%, respectively). Similarly, considering age as an exposure parameter, the delirium incidence moved from 0% in participants < 60 to 35% in patients over 90 years old (Table 2). Using the same criteria, variables associated with an incidence of delirium higher than the fixed threshold of significance (25%) were dementia (37%), parkinsonism (30%), psychiatric diseases (32%), chronic treatment with typical antipsychotics (40%), atypical antipsychotics (43%) and antiepileptics (32%) (Table 2). A high incidence of delirium was also observed in patients who received nasogastric tubes (44%) and urinary catheters (31%) (Table 2) during hospitalization. Regarding outcomes, delirium incidence was also higher than the average of the population in patients discharged to nursing homes (33%) and in participants who subsequently died during hospital stay (35%) (Table 2).

**Table 2:** Overview of the frequency of delirium in the studied population, considering each parameter in a separate way (frequency ≥ 30% is considered significant, with respect to the average of the population, and indicated in bold)

Parameter	Prevalence of the parameter	Frequency of delirium in the group with the parameter	Cases of delirium with the parameter vs all cases of delirium
**Age and frailty**			
Age > 85 years old	44%	27%	59%
Age > 90 years old	14%	**35%**	32%
CFS > 4	57%	29%	85%
CFS > 5	36%	**34%**	62%
CFS > 6	18%	**38%**	35%
**Drugs and diseases**			
Chronic illnesses > 2	87%	20%	86%
Hypertension	69%	19%	67%
Cardiomyopathy	47%	16%	36%
Arrythmia	36%	21%	38%
Anemia	25%	11%	14%
COPD	30%	16%	24%
Diabetes	27%	16%	22%
Obesity	8%	11%	4%
Dyslipidemia	20%	13%	13%
Parkinsonism	9%	**30%**	13%
Previous stroke	14%	24%	17%
CKD	16%	16%	13%
Cancer	26%	12%	15%
Dementia	29%	**37%**	55%
Psychiatric disease other than dementia	18%	**32%**	38%
Peripheral artery disease	29%	22%	31%
Liver disease	12%	18%	11%
Gastrointestinal disease	25%	16%	19%
CIRS Comorbidity Score > 8	80%	19%	77%
CIRS Severity Index > 2	33%	15%	25%
Chronic drug treatments > 5	63%	19%	60%
α-blockers	14%	25%	17%
Typical antipsychotics	2%	**40%**	3%
Atypical antipsychotics	12%	**43%**	26%
Benzodiazepines	21%	25%	26%
Antiepileptics	9%	**32%**	14%
Acetaminophen	7%	26%	9%
Central anticholinergic drugs	< 1%	**33%**	< 1%
**Conditions occurring during the hospital stay**			
Nasogastric tube insertion	3%	**44%**	7%
Urinary catheter insertion	31%	**31%**	49%
Use of other invasive device	2%	27%	3%
**Discharge setting**			
Home	77%	16%	62%
Nursing home	11%	**33%**	18%
Hospital death	11%	**35%**	19%

The matrix of interdependence of clinical characteristics with a higher frequency in patients with delirium than in other study participants is shown in Supplementary Table 1. Notably, 71% of participants aged > 85 had CFS > 4. Therefore, the group of patients with CFS > 4 or age > 85 years old, corresponding to 70% of the studied population, included 91% of cases of delirium, while patients not belonging to this group had a frequency of delirium well below the average of the population (6% vs 20%). The use of invasive devices was another parameter highly associated with delirium (Table 2), but not exhibit a high degree of interdependence with CFS and age (Supplementary Table 1). The group of patients with at least one feature among age > 85 years old, CFS > 4 and use of invasive devices during hospital stay, corresponding to 72% of the studied population, included 95% of the observed cases of delirium. Conversely, patients not belonging to this group had a frequency of delirium of just 4%.

In addition to frailty and age, we also considered the coexistence of parameters occurring with high frequency in the population and their incidence of delirium. Basing on the interdependency matrix shown in Supplementary Table 1, we identified three categories of parameters: comorbidities (dementia and psychiatric diseases), chronic pharmacologic treatments (antipsychotics) and insertion of invasive devices during the stay. The analysis of the correlation of the coexistence of at least two (model 1) or three (model 2) of the considered parameters with delirium is shown in Table 3. Specifically, two-parameter correlation analysis, combining the parameters of Table 3, model 1, in pairs, showed that the incidence of delirium was more than doubled for the pair “antipsychotic treatment and insertion of invasive devices” (50%). The inclusion of dementia as a third parameter of selection, implying the reduction of the group of participants with the selected characteristics to only 5% of the entire population, retrieved an incidence of delirium of 57%. Similarly, the coexistence of age > 85 years old, antipsychotic treatment and insertion of invasive devices, occurring in only 3% of participants, was associated with a record 61% incidence of delirium (Table 3, model 3).

**Table 3:** Analysis of correlation between the coexistence between clinical parameters, selected based on the findings of Table 2, and incidence of delirium

Parameters	Number (% of the total population)	Incidence of delirium	Cases of delirium with the condition vs all cases of delirium
Model 1—Two-parameter correlation analysis (antipsychotic treatment, insertion of invasive devices, dementia, psychiatric diseases)			
Antipsychotic treatment and insertion of invasive devices	32 (5%)	50%	14%
Dementia and antipsychotic treatment	60 (10%)	47%	24%
Dementia and insertion of invasive devices	80 (14%)	45%	31%
Psychiatric diseases and antipsychotic treatment	36 (6%)	44%	14%
Psychiatric diseases and insertion of invasive devices	40 (7%)	48%	16%
Model 2—Three-parameter correlation analysis (antipsychotic treatment, insertion of invasive devices, dementia, psychiatric diseases)			
Dementia, antipsychotic treatment and insertion of invasive devices	28 (5%)	57%	14%
Psychiatric diseases, antipsychotic treatment and insertion of invasive devices	15 (3%)	60%	8%
Model 3—Three-parameter correlation analysis (with age)			
Age > 85, antipsychotic treatment and insertion of invasive devices	18 (3%)	61%	9%

Delirium was classified as hyperactive in 45%, hypoactive in 31% and mixed in 24% of cases. As shown in Supplementary Table 3, among the studied parameters, CFS > 5, dementia, use of antipsychotic drugs and invasive devices were mainly associated with hypoactive delirium. Lethality was also markedly higher in hypoactive (32%) than hyperactive delirium (9%), in comparison with the mean value of the sample (i.e. 11%). These data suggest that the excess lethality (+ 208%) associated with delirium could be univocally determined by the hypoactive form.

## Discussion

In a large group of older patients hospitalized in an acute internal medicine and geriatric ward, more than 95% of those who experienced delirium during hospital stay had at least one among age > 85 years old, CFS > 4 and insertion of invasive devices such as urinary catheters or nasogastric tubes. At the individual patient’s level, the main factors maximizing the risk of experiencing delirium during the stay were dementia, psychiatric pathologies, chronic assumption of antipsychotic drugs, and use of invasive devices.

To date, clinical studies and meta-analyses on factors associated with delirium in older people have been mainly focused on specific care settings, especially post-surgery or palliative care, where triggers are standardized and easily identifiable [30, 31]. Even in the general acute-care setting, however, the existing literature shows associations between delirium and a large number of factors related to demographical, functional and social characteristics of patients, previous illnesses, chronic comorbidities and pharmacologic treatments [32]. In this context, the stratification of the risk of delirium on hospital admission may be challenging from a clinical perspective, for the difficulties in identifying a small number of robust predictors that could be used for screening purposes and personalization of geriatric care.

Recently, frailty has emerged as a major factor associated with delirium. In a nationwide point-prevalence study conducted in Italy, each 0.1 increase in the FI score was associated with a 65% increase in the odds of delirium during acute hospitalization [14]. In a recent multicenter study conducted in the UK, increasing CFS scores were associated with both increased prevalence and reduced recognition of delirium during hospitalization [33]. In frail individuals, delirium can also represent an atypical manifestation of acute illnesses, as suggested by studies conducted during the COVID-19 pandemic [34]. The relationship between delirium and frailty seems also to be independent of the tools used for detecting frailty [13]. For this reason, the inclusion of frailty assessment ameliorated the performance of delirium clinical prediction tools in the Intensive Care Unit (ICU) setting [35, 36].

The occurrence of delirium in frail patients is also predictive of worse clinical outcomes. Although according to some studies, delirium-associated mortality tended to be higher in fit than in frail individuals [37], in geriatric settings such as postoperative care after hip fracture, the co-occurrence of frailty and delirium resulted in poorer functional status at discharge [38] and higher risk of dysphagia [39]. In this regard, delirium itself can be considered as a marker of frailty [40].

The frailty concept, however, encompasses several clinical, functional and social factors identified by previous meta-analyses as associated with the risk of delirium [12]. For example, the CIRS scale, originally developed for estimating the burden of comorbidity in older patients, is strongly correlated to measures of frailty, such as the CFS or the FI [41], especially in individuals where frailty is a consequence of diseases rather than age-related [42]. In geriatric medicine, the frailty paradigm allows to capture the complexity of older patients, in which several conditions, influencing the risk of adverse outcomes including delirium, may consistently overlap [43]. This is the main reason for the methodology adopted in the present investigation, that accounted for the high probability of interdependency between the clinical, demographical and pharmacological parameters considered in a population of geriatric patients. CFS score > 4, in particular, exhibited a consistent frequency of overlap with many of the other parameters considered in the investigation, including chronic illnesses and pharmacological treatments. So, the association between these factors and delirium, demonstrated in previous studies, may not be independent of frailty [44].

The results of the present investigation also reinforce the importance of avoiding unnecessary insertion of invasive devices, such as nasogastric tubes or urinary catheters, for reducing the risk of delirium. In patients hospitalized in acute-care wards, indwelling urinary catheters are in fact associated with a consistent increase in the risk of delirium [45]. Invasive procedures, including also the insertion of a nasogastric tube, have been recently recognized as a significant predictor of delirium in older individuals admitted to a cardiac ICU dedicated to older patients [46].

A diagnosis of dementia and usual treatment with antipsychotic drugs were instead recognized as the individual factors maximizing the risk of delirium in each patient. Delirium superimposed on dementia is a distinct clinical entity that is often underdiagnosed in older individuals, due to difficulties in disentangling the clinical manifestations of delirium from the usual presentation of dementia [47]. It is generally associated with a poor outcome and consistent risk of nursing home admission, loss of autonomy and further cognitive decline [48]. A recent study also highlighted that neurosensorial multimorbidity, i.e. the presence of preexisting cognitive decline or dementia with visual or hearing deficits, is one of the main predisposing factors of delirium [49].

Antipsychotic drugs are often used to prevent and treat episodes of hyperactive delirium in older patients hospitalized in internal medicine wards [50]. The use of these drugs for delirium treatment is controversial, promoting, at most, sedation, but not influencing the delirium duration and outcome in a favorable way [50, 51]. Many older patients, however, may chronically take antipsychotic drugs for several reasons, including Behaviour and Psychological Symptoms of Dementia (BPSD) [52, 53]. Such prescriptions are associated with an increased risk of delirium, especially in those already suffering from dementia [54]. Therefore, the results of the present study support the concept that patients admitted to acute-care hospital wards who chronically take antipsychotic drugs are at increased risk of delirium, irrespective of the presence of dementia.

Some limitations should be considered when interpreting our findings. First, the retrospective study design did not allow comprehensive investigations of outcomes and a systematic detection of delirium duration. According to the existing literature the cases of delirium not detected during hospital stay and established a posteriori after review of case notes should be classified as a distinct entity called “unrecognized delirium” or “suspected delirium”, not equivalent to delirium diagnosed by CAM [24]. The absence of this categorization in our study could have led to an overestimation of delirium cases in frailer patients. However, the methodology of retrospective delirium detection was considered acceptable in previous studies [25, 26], and the outcomes of subjects with recognized and unrecognized delirium were substantially similar in a nationwide investigation conducted in the UK [24]. Furthermore, the enrolment of patients admitted to just one clinical center might have influenced the generalizability of results to other settings. The use of CFS instead of FI might also have limited a precise estimate of the severity of frailty in each patient. These limitations, however, are counterbalanced by the comprehensiveness of clinical data collected for each participant and by the statistical approach adopted in the investigation, that allowed to account for the interdependency between several clinical variables and frailty. This approach led to the identification of a limited number of clinical variables showing a strong association with delirium, for the first time with great clarity.

## Conclusions

In a group of older patients acutely hospitalized in an acute-care high-turnover ward, delirium almost exclusively occurred in participants with one among age > 85 years old, CFS > 4 and presence of indwelling invasive devices. In this population, dementia, chronic antipsychotic drug treatment, and the use of invasive devices during hospital stay were the main factors maximizing the incidence of delirium at the individual level. Such results could represent the basis for the development of clinical prediction tools able to identify patients at risk of delirium in heterogeneous groups of patients admitted to acute-care geriatric services.

### Supplementary Information

Below is the link to the electronic supplementary material.

Supplementary file 1 (DOCX 23 KB)

## Data Availability

Data are available in anonymous form upon reasonable request addressed to the corresponding author.
