# Supplementary material for: Factors associated with delirium in a real-world acute-care setting: analysis considering the interdependence of clinical variables with the frailty syndrome
Source: Eur Geriatr Med. 2024 Feb 8;15(2):411–21. doi: 10.1007/s41999-024-00934-x (PMC10997727; doi:10.1007/s41999-024-00934-x)
Supplement: Supplementary file 1 — (DOCX 23 KB) [file 41999_2024_934_MOESM1_ESM.docx]

**SUPPLEMENTARY TABLE 1**

Matrix of interdependence of exposure variables, selected considering the excess frequency of delirium detailed in Table 2. Parameters with a low degree of interdependence (<0.50) are indicated in bold.

|  | **CFS >4** | **CFS >6** | **Age >85** | **Age >90** | **Dementia** | **Psychiatric diseases** | **Parkinsonism** | **Antipsychotics (typical/atypical)** | **Antiepileptics** | **Invasive devices** | **Hospital death** | **Nursing Home** |
| --- | --- | --- | --- | --- | --- | --- | --- | --- | --- | --- | --- | --- |
| **CFS >4** | - | - | 0.71 | 0.84 | 0.99 | 0.73 | 0.88 | 0.90 | 0.78 | 0.77 | 0.76 | 0.78 |
| **CFS >6** | - | - | 0.56 | **0.33** | 0.70 | **0.26** | **0.38** | 0.51 | **0.28** | 0.53 | **0.40** | **0.28** |
| **Age >85** | - | - | - | - | 0.57 | **0.38** | **0.36** | 0.51 | **0.38** | 0.52 | 0.55 | 0.50 |
| **Age >90** | - | - | - | - | **0.48** | **0.14** | **0.14** | **0.25** | **0.14** | **0.45** | **0.20** | **0.22** |
| **Dementia** | - | - | - | - | - | 0.58 | 0.66 | 0.79 | **0.46** | **0.47** | **0.44** | **0.45** |
| **Psychiatric diseases** | - | - | - | - | - | - | **0.36** | **0.47** | **0.36** | **0.39** | **0.13** | **0.20** |
| **Parkinsonism** | - | - | - | - | - | - | - | **0.26** | **0.14** | **0.40** | **0.10** | **0.16** |
| **Antipsychotics (typical/atypical)** | - | - | - | - | - | - | - | - | **0.28** | **0.42** | **0.24** | **0.23** |
| **Antiepileptics** | - | - | - | - | - | - | - | - | - | **0.36** | **0.04** | **0.12** |
| **Invasive devices** | - | - | - | - | - | - | - | - | - | - | 0.58 | **0.47** |
| **Hospital death** | - | - | - | - | - | - | - | - | - | - | - | - |
| **Nursing home** | - | - | - | - | - | - | - | - | - | - | - | - |

**SUPPLEMENTARY TABLE 2**

Comparison of the frequency of use of the main drug classes before hospital admission in patients who presented features of delirium and those who did not.

| **DRUG CLASSES** | **PATIENTS WITH DELIRIUM (n=117)** | **PATIENTS WITHOUT DELIRIUM (n=470)** |
| --- | --- | --- |
| ACE-I, % | 21 | 26 |
| ARB, % | 18 | 19 |
| Calcium antagonists, % | 22 | 23 |
| β receptor blockers, % | 43 | 51 |
| α receptor blockers, % | 17 | 13 |
| Digoxin, % | 3 | 5 |
| Other antiarrhytmics, % | 5 | 5 |
| Aspirin, % | 39 | 41 |
| Anticoagulants, % | 24 | 26 |
| Allopurinol, % | 9 | 13 |
| SSRI/SNRI, % | 24 | 22 |
| Typical antipsychotics, % | 3 | 1 |
| Atypical antipsychotics, % | 25 | 9 |
| Benzodiazepines, % | 26 | 20 |
| Antiepileptics, % | 14 | 7 |
| Loop diuretics, % | 31 | 40 |
| Thiazide diuretics, % | 6 | 18 |
| Potassium sparing diuretics, % | 10 | 16 |
| PPIs, % | 44 | 56 |
| Acetaminophen, % | 9 | 7 |
| Opioid analgesics, % | 10 | 10 |
| NSAIDs, % | 2 | 2 |
| Cholinesterase inhibitors, % | 1 | 0 |
| Anticholinergic agents, % | 1 | 1 |
| Vasodilators, % | 3 | 5 |
| Corticosteroids, % | 6 | 11 |

ACE-I=Angiotensin Converting Enzyme Inhibitors; ARB=Angiotensin Receptor Blockers; SSRI=Selective Serotonin Reuptake Inhibitors; SNRI=Selective Norepinephrine Reuptake Inhibitors; PPIs=Proton Pump Inhibitors; NSAIDs=Non-Steroidal Anti-Inflammatory Drugs.

**SUPPLEMENTARY TABLE 3**

Correlations between the clinical parameters identified in the main analysis as correlated with delirium subtypes (hyperactive and hypoactive). The percentages indicate the frequency of hyperactive and hypoactive delirium, respectively in column 2 and 4, with the condition identified by clinical parameters respect with the population with the specific delirium subtype. Instead, in column 3 and 5 the frequency of hyperactive and hypoactive delirium in the population with the parameter is reported, compared with the mean value MV of the relative delirium subtype in brackets.

| **PARAMETERS** | **Hyperactive delirium**  **(MV 9%)** | **Hyperactive delirium in the population with the parameter (respect with MV value)** | **Hypoactive delirium**  **(MV 6.3%)** | **Hypoactive delirium in the population with the parameter**  **(respect with MV value)** |
| --- | --- | --- | --- | --- |
| CFS>5 | 53% | 13.2 (+46%) | 70% | 12.2% (+94%) |
| Dementia | 38% | 13.2 (+28%) | 78% | 12.2% (+170%) |
| Psychiatric diseases | 26% | 13.2 (+49%) | 24% | 8.7% (+38%) |
| Antipsychotic drug treatment | 19% | 12.9 % (+46%) | 35% | 8.7% (+172%) |
| Use of invasive devices | 42% | 11.9 % (+46%) | 57% | 11.3% (+80%) |
| Death or nursing home admission | 32% | 13.5% (+50%) | 43% | 12.7% (+102%) |
| Death | 9% | 8.1% (-11%) | 32% | 19.3% (+208%) |
